# Study of Localized Corrosion Susceptibility of Ni-Based Superalloys Employing Electrochemical Noise Technique

**DOI:** 10.3390/ma19112424

**Published:** 2026-06-05

**Authors:** Facundo Almeraya-Calderon, Miguel Sergio Huerta-Zavala, Erick Maldonado-Bandala, Demetrio Nieves-Mendoza, Jesus Manuel Jaquez-Muñoz, Miguel Angel Baltazar-Zamora, Laura Landa-Ruiz, Francisco Estupinan-Lopez, Javier Olguin-Coca, Juan Pablo Flores-De los Rios, Citlalli Gaona-Tiburcio

**Affiliations:** 1 Universidad Autónoma de Nuevo León, FIME, Centro de Investigación e Innovación en Ingeniería Aeronáutica (CIIIA), San Nicolás de los Garza 66455, Mexico; facundo.almerayacld@uanl.edu.mx (F.A.-C.); miguel.huertaz@uanl.edu.mx (M.S.H.-Z.); francisco.estupinanlp@uanl.edu.mx (F.E.-L.); 2 Facultad de Ingeniería Civil, Universidad Veracruzana, Xalapa 91000, Mexico; erimaldonado@uv.mx (E.M.-B.); dnieves@uv.mx (D.N.-M.); mbaltazar@uv.mx (M.A.B.-Z.); lalanda@uv.mx (L.L.-R.); 3 Instituto Tecnológico de Ciudad Juárez, Metal-Mechanics Department, Av. Tecnológico 1340, Ciudad Juárez 32500, Mexico; 4 Área Académica de Ingeniería y Arquitectura, Universidad Autónoma del Estado de Hidalgo, Carretera Pachuca-Tulancingo Km. 4.5, Hidalgo 42082, Mexico; olguinc@uaeh.edu.mx; 5 Instituto Tecnológico de Chihuahua, Tecnológico Nacional de Mexico, Av. Tecnologico 2909, Chihuahua 31130, Mexico; jpfloresr@itch.edu.mx

**Keywords:** localized corrosion, electrochemical noise, pitting, cyclic potentiodynamic polarization

## Abstract

Inconel superalloys are employed in demanding components of different equipment. However, they can be exposed to atmospheric corrosion systems, such as marine and industrial environments. This research is focused on studying the localized corrosion susceptibility of Inconel 600, 690 and 718 exposed to H_2_SO_4_, 1 wt.% and 3.5 wt. % NaCl solutions, simulating marine and industrial atmospheres at 25 ± 0.5 °C. Localized corrosion behavior was characterized by electrochemical noise (EN) and cyclic potentiodynamic polarization (CPP) curves according to ASTM 6-199 ASTM G61 standards. The EN technique was analyzed through time series and analysis for chaotic systems, such as Hurst, Lyapunov and Husdorff coefficients, to determine the corrosion type of each system to reduce the uncertainty in common statistical analysis. The EN results show how Inconel superalloys tend to present localized attacks, being more notorious in NaCl. The application of specialized methods such as Hurst and Lyapunov helped to determine the corrosion system when alloys were characterized by EN. The results indicated that all superalloys exhibit positive hysteresis under CPP, indicating susceptibility to localized pitting corrosion.

## 1. Introduction

Superalloys are materials designed for high-temperature applications, characterized by outstanding resistance to mechanical and chemical deterioration at temperatures approaching their melting points [1]. Since their introduction in the first half of the twentieth century, these alloys have played a transformative role in advanced engineering. A notable example is their use in aero-engines that power modern commercial aircraft. Superalloys are utilized in the hottest regions of turbine engines, where components are subjected to extreme loads, making the structural integrity of these parts critically important. In fact, the evolution of superalloys has been closely intertwined with the development of jet engines, as modern aircraft would be incapable of flight without them. Continuous efforts are being made to enhance their temperature capabilities, enabling engines to power aircraft such as the Airbus A380 and the Boeing 787 Dreamliner [2,3,4,5].

Materials employed in the hot sections of jet engines must maintain high performance levels and structural stability over long service periods while operating under severe mechanical stresses and corrosive environments [6]. Superalloys constitute a vital class of high-temperature materials used in the manufacture of gas turbines, aircraft engines, rocket components, and equipment for the petroleum industry. These alloys are well suited for extreme operating conditions and can retain their mechanical strength at temperatures ranging from 1200 to 1400 °C for extended durations.

Currently, superalloys are commonly defined as a group of austenitic alloys, which are classified into three main categories based on their primary elements: nickel-based, cobalt-based, and nickel–iron-based alloys. Nickel-based superalloys exhibit superior high-temperature strength and creep resistance compared to the other types, making them suitable for the most demanding applications. Nickel–iron superalloys are known for their good ductility and toughness, while cobalt-based superalloys offer enhanced resistance to high-temperature corrosion [1].

Several conventional electrochemical methods have been widely applied to evaluate corrosion kinetics and elucidate reaction mechanisms, including potentiodynamic polarization (PP), electrochemical impedance spectroscopy (EIS), and linear polarization resistance (LPR). Nevertheless, these techniques involve the application of external electrical signals, which may disturb the electrochemical system during measurements [7,8]. In contrast, the electrochemical noise (EN) technique has gained increasing attention in recent years for corrosion investigation and monitoring, offering significant advantages for corrosion science. One of its key benefits is its capability to detect and analyze the early stages of localized corrosion.

Electrochemical noise refers to the spontaneous, low-amplitude fluctuations in potential and current that naturally occur during electrochemical processes. Throughout corrosion, mainly anodic and cathodic reactions generate small transient charge variations at the electrode surface. These fluctuations appear as potential and current noise, which can be analyzed and interpreted using corrosion maps [9,10]. The observed transients are associated with anodic and cathodic events arising from both stochastic processes, such as passive film breakdown and repassivation, and deterministic processes, including pit initiation and growth [11,12].

Xia et al. [13] reports a variety of mathematical approaches and parameters that can be applied to electrochemical noise (EN) data to determine corrosion types and estimate corrosion rates. Among these, the most widely used methods are those based on the time domain such as statistical analyses, including noise resistance, skewness, kurtosis, and the localization index (LI), as well as chaos analysis, recurrence quantification analysis, and fractal analysis. In addition, frequency-domain techniques are widely employed, including shot noise analysis, Fast Fourier Transform (FFT) for power spectral density evaluation, and noise impedance measurements. Furthermore, several time–frequency domain methods have been developed, such as the Hilbert–Huang transform, discrete wavelet transform, Stockwell transform, and related techniques [14,15,16].

Based on various chaos theories, the Hurst exponent (H) can be calculated to quantify the level of autocorrelation within a data series and to evaluate how past events influence future behavior. This parameter was first introduced by Hurst in the analysis of hydrological data, specifically to aid in the design of a dam on the Nile River [15,16,17]. Later, through the contributions of Mandelbrot and Wallis [18], the Hurst exponent became a widely used mathematical tool and was successfully applied across numerous scientific disciplines.

The aim was to study the susceptibility to localized corrosion of Inconel 600, 690, and 718 exposed to 1 wt. % H_2_SO_4_, and 3.5 wt. % NaCl solutions at 25 ± 0.5 °C, using electrochemical noise (EN) and cyclic potentiodynamic polarization (CPP) curves according to ASTM 6-199 and ASTM G61 standards. The electrochemical characterization of superalloys is of significant interest due to their potential applications in the aeronautical industry, particularly in components such as turbine blades and landing gear. Structural aircraft elements manufactured from superalloys are exposed to a variety of aggressive environments, including industrial atmospheres characterized by acid rain (H_2_SO_4_), which is formed from the chemical reactions of sulfur dioxide and nitrogen oxides found in the atmosphere with water and chemical contaminants that result in nitric and sulfuric acids and marine environments rich in chloride ions (typical concentration of sea water 3.5% NaCl). Under these conditions, especially when aircraft remain on the ground, superalloys are vulnerable to low-temperature pitting corrosion [19,20].

## 2. Materials and Methods

### 2.1. Materials

The materials used were commercial superalloys in the form of cylindrical bars 0.5” in diameter. They were employed and tested in the as-received condition.

INCONEL ^®^ 600 Special Metals Corporation, Huntington, WV, USA (nickel–chromium–iron) alloy is a standard engineering material for applications requiring corrosion and heat resistance. The alloy also exhibits excellent mechanical properties, offering an ideal combination of high strength and good workability. INCONEL^®^ 690 (Special Metals Corporation, Huntington, WV, USA) is a high-chromium–nickel alloy with excellent resistance to various corrosive aqueous media and high-temperature atmospheres. INCONEL^®^ 718 (Special Metals Corporation, Huntington, WV, USA) is a high-strength, corrosion-resistant nickel–chromium material. Its good tensile strength, fatigue resistance, creep resistance, and fracture resistance have led to its use in a wide range of applications. These superalloys are found in rocket components, turbine engine rings and housings, petrochemical processing furnaces, and cryogenic tanks.

The chemical composition of both superalloys is presented in Table 1 and was determined via X-ray fluorescence.

### 2.2. Microstructural Characterization

The superalloys were prepared via metallographic techniques following ASTM E3-95 [21], using a series of silicon carbide abrasives with grit sizes ranging from 80 to 4000, polished with 0.1-micron alumina. The microstructure was revealed using a solution of 5 mL H_2_SO_4_, 3 mL HNO_3_, 92 mL HCl. The microstructure of superalloys was obtained using optical microscopy (OM, Olympus, Hamburg, Germany). Finally, to analyze the surfaces of the corrosion-tested samples, a microscopic analysis was performed using scanning electron microscopy (SEM; JEOL-JSM-5610LV, Tokyo, Japan) at 50 and 500× magnification with a secondary electron (SE) detector. The chemical composition of the surface elements was determined using energy-dispersive X-ray spectroscopy (EDS).

### 2.3. Electrochemical Characterization

The corrosion behavior of the superalloys was determined using electrochemical tests, including cyclic potentiodynamic polarization curves and electrochemical noise. The samples were exposed to two electrolytes, 1 wt. % H_2_SO_4_ and 3.5 wt. % NaCl solutions (the electrolytes were not de-aerated), at room temperature (25 ± 0.5 °C), with an exposed working area of 1 cm^2^. The samples were polished with SiC grit paper till 600 grade, followed by ultrasonic cleaning in ethanol and deionized water for about 10 min each. All results were obtained in triplet. The equipment used was a Potentiostat/Galvanostat/ZRA (Zero Resistance Ammeter) (Solartron 1287A, Bognor Regis, UK).

Electrochemical measurements were performed as follows: CPP curves were obtained according to ASTM G61 [22,23], with a potential sweep of ±700 mV from the equilibrium potential at a polarization rate of 60 mV/min. The tests were performed using a three-electrode cell [Inconel superalloys served as working electrodes (WEs), a saturated calomel reference electrode (RE) was used, and a platinum mesh was used as the counter electrode (CE)].

**Figure 1:**
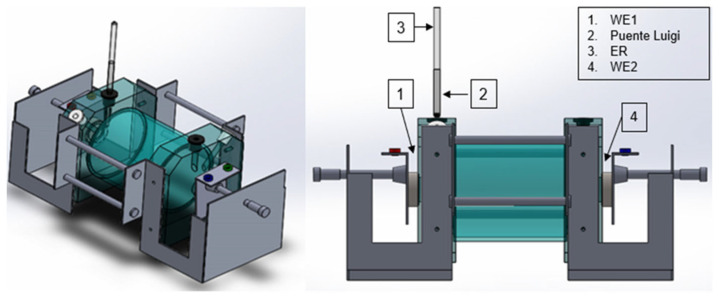
Three-electrode cell for electrochemical noise (EN) measurements [27].

Electrochemical noise (EN) measurements were performed according to ASTM G199-09 [24], which enables the determination of the noise resistance (R_n_) and corrosion rate in corrosive environments. In each test (Figure 1), two nominally identical specimens were employed as working electrodes (WE1 and WE2), along with a saturated calomel electrode serving as the reference electrode (RE). Electrochemical current noise (ECN) was recorded through the galvanic coupling between the two identical working electrodes, while electrochemical potential noise (EPN) was simultaneously measured between one working electrode and the reference electrode. Both current and potential noise signals were monitored for each electrode–electrolyte system under open-circuit conditions. Each EN dataset consisted of 4096 data points per test at an acquisition rate of 1 data point per second. The time-domain signals of current and potential were visually examined to identify transient events and to assess the temporal evolution of fluctuation frequency and amplitude [25,26].

The electrochemical noise (EN) signal, after the DC (DC) component was removed using a ninth-degree polynomial, was analyzed; this polynomial degree was selected due to the 5th and 7th EN signal still presenting DC, so to eliminate the signal, a 9th polynomial filter was the best option for those materials. Statistical parameters, including noise resistance (Rn), localization index and skewness, were then calculated from the detrended signals. Hurst, Lyanupov, and Hausdorff coefficients were calculated using a Python (programming language version 3.13.1) program based on nolds package.

For chaotic analysis, the embedding dimension selected was of 5 for all the signals with a false nearest neighbors method, with a factor of error lower than 5%. The time delay employed was of 3 based on array manifold interpolation (AMI) to avoid correlation between the reconstructed coordinates.

Electrochemical noise signals associated with localized corrosion are inherently non-stationary due to the stochastic nature of pit nucleation, growth, and repassivation processes. In the present study, strict stationarity cannot be assumed; therefore, signal stationarity was approximated through polynomial detrending combined with segmented time-window analysis. The EN time series were divided into consecutive 512 s windows, which reduces the influence of slow drift while preserving transient electrochemical events associated with localized corrosion. Although the total signal length (4096 s) is shorter than that typically required for fully converged nonlinear metrics, this windowed approach enables relative comparison of nonlinear parameters and assessment of their temporal stability. The robustness of the Hurst exponent and Lyapunov exponent is supported by the persistence of material-dependent trends across successive windows, rather than reliance on isolated values. Nevertheless, it is acknowledged that short time series may introduce uncertainty in absolute parameter estimation, particularly for the Hausdorff dimension, which is more sensitive to data length and scaling range. For this reason, nonlinear parameters are interpreted here as comparative and mechanistic indicators, supported by statistical EN descriptors and independent electrochemical techniques, rather than as standalone quantitative metrics.

## 3. Results and Discussion

### 3.1. Microstructures of Superalloys by OM

**Figure 2:**
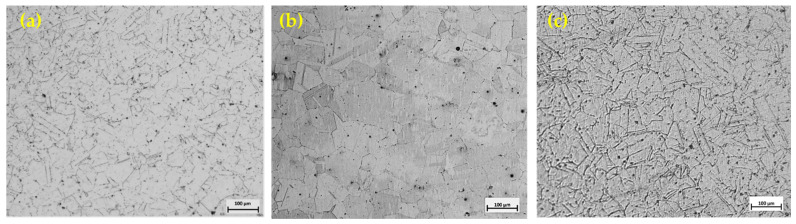
OM microstructures of superalloys (initial conditions): (**a**) Inconel 600, (**b**) Inconel 690 and (**c**) Inconel 718.

The microstructures of superalloys were obtained via optical microscopy. In Figure 2a, the microstructure of Inconel 600 consists primarily of an austenitic (γ) nickel–chromium matrix, characterized by a face-centered cubic (FCC) solid solution. It is a non-heat-hardenable alloy, reinforced by solid solution with carbides (generally Cr_7_C_3_ o Cr_23_C_6_) precipitated at the grain boundaries. Figure 2b shows that the microstructure of Inconel 690 is a nickel-based solid solution (phase γ) with a high chromium content, characterized by a face-centered cubic (FCC) austenitic matrix. It exhibits excellent metallurgical stability, with homogeneous grain and chromium carbides (mainly Cr_23_C_6_) dispersed at the grain boundaries for high corrosion resistance, and Figure 2c shows the microstructure of Inconel 718 is that of a nickel-based superalloy with an austenitic (γ) of nickel–iron base matrix, reinforced primarily by phase precipitates that are (γ″ (Ni_3_Nb) tetragonal and, to a lesser extent (γ′(Ni_3_(Al,Ti), cubic. This high-performance alloy is characterized by the presence of δ(delta) phases and carbides (CM), key to grain size control and creep resistance, which is optimized through various treatments.

### 3.2. Electrochemical Noise

#### 3.2.1. Time-Domain Analysis

Electrochemical noise is used to assess material corrosion activity by simultaneously recording spontaneous potential and current fluctuations during the corrosion process. The EN signal consists of three components: a random component, a steady-state component, and a direct current component.

To calculate noise resistance (Rn), the standard deviation from time-series data must be determined. These statistical parameters offer insights into corrosion mechanisms and kinetics. Turgoose and Cottis [28] revealed that higher corrosion rates correspond to increases in variance and standard deviation. Use Equation (1) to compute the standard deviation (σ), standard deviation of the EPN data (σv), standard deviation of the ECN data (σI), and working electrode area (A) and derive R_n_ (see Equation (2)).

(1) $$\sigma_{x}=\sqrt{\bar{x^{2}}}=\sqrt{\frac{\sum_{1}^{N}{\left(x_{i}-\bar{x}\right)}^{2}}{N}\;\;\;\;\;\;\;}$$

(2) $$R_{n}=\frac{\sigma_{v}}{\sigma_{I}}*A$$

The electrochemical noise resistance (R_n_) and linear polarization resistance (LPR or R_p_) are related; the Stern–Geary equation [29] (see Equation (3)) can be used as an analog relation to determine corrosion kinetics. B is a constant with a recommended value of 0.026 V for active and 0.052 V for passive corrosion.

(3) $$i_{corr}=\frac{B}{R_{n}}$$

The localization index has limitations, as noted by Mansfeld and Sun [30], and, therefore, its information should be used with caution. For this reason, skewness should be considered when determining the type of corrosion. However, in the patent of Reid and Eden [31], they indicate that the third and fourth statistical moments, respectively, skewness (see Equation (4)), can be used to determine the type of corrosion [12,30,31,32], where N is the number of data studied and x is the EN signal.

(4) $$skewness=\frac{1}{N}\sum_{i=1}^{N}\frac{{{(x}_{i}-\bar{x})}^{3}}{\sigma^{3}}$$

Table 2 shows the relationship between the skewness values used to determine the material’s corrosion type.

**Figure 3:**
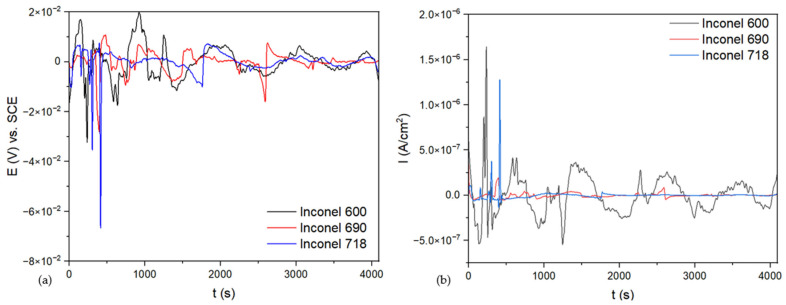
Electrochemical potential (**a**) and current (**b**) noise time series for the alloys exposed to 3.5 wt. % NaCl solutions.

Figure 3 shows the time series of potential and current for the alloys exposed to NaCl. In the media, Inconel 600 and Inconel 718 exhibited high-amplitude transients in potential and current before 500 s. However, Inconel 718 showed a reduction in transient amplitude. On the other hand, Inconel 600 presented oscillations in potential and current, indicating an increase in corrosion kinetics. Table 3 shows that Inconel 600 had lower noise resistance (29,717 Ω·cm^2^), while Inconel 690 had higher noise resistance. All the samples presented localized corrosion values, with Inconel 718 showing the highest value (0.71), indicating that the localized corrosion process tends to be more aggressive.

**Figure 4:**
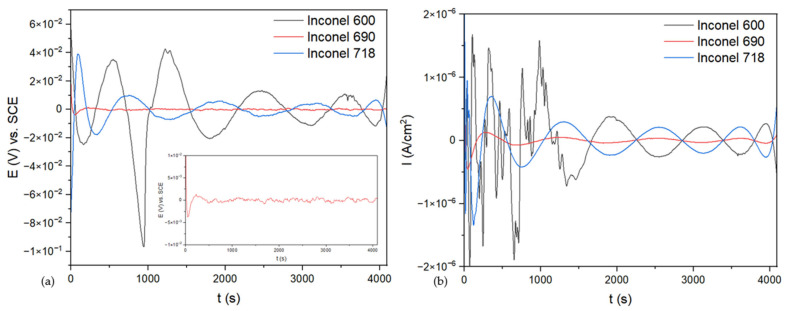
Electrochemical potential (**a**) and current (**b**) noise time series for the alloys exposed to 1 wt. % H_2_SO_4_ solution.

Figure 4 shows the EPN and ECN time series of superalloys exposed to H_2_SO_4_. Both samples presented fluctuating behavior. Inconel 600 presented an amplitude in current of 4 × 10^−6^ A/cm^2^ and reduced the amplitude as time advanced, indicating that the corrosion kinetics decrease as time advances. All the samples presented similar behaviors. It is important to mention that Inconel 690 presented 6080 Ω·cm^2^, being the least resistant to noise; Inconel 600 presented 42,979 Ω·cm^2,^ being the most resistant to corrosion (see Table 4). Also, the samples showed values indicative of localized corrosion.

**Figure 5:**
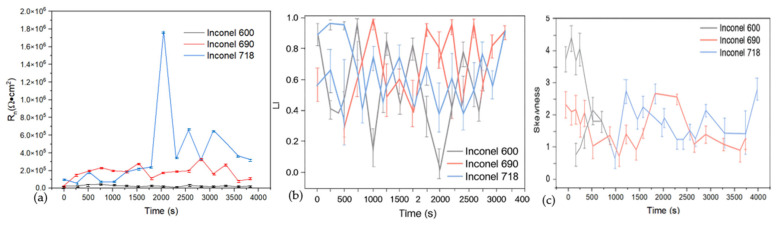
Variation in Rn (**a**), LI (**b**) and skewness (**c**) in different time lapses (each 512 s) in 3.5 wt. % NaCl solution.

Figure 5 shows the variation in parameters Rn (Figure 5a), LI (Figure 5b), and skewness (Figure 5c) over different time lapses (every 512 s). The Inconel 718 showed an increase in R_n_ at 2048 s, which may be associated with a reduction in corrosion kinetics; the other samples showed similar behavior. The LI ranged from 0.2 to 1; this indicates that the alloys exhibited localized corrosion in all samples. The skewness presented values associated with pitting in the first stage; however, the value of skewness decreases as time advances. It can occur when a localized process begins to occur on the entire surface, generalizing the attack.

**Figure 6:**
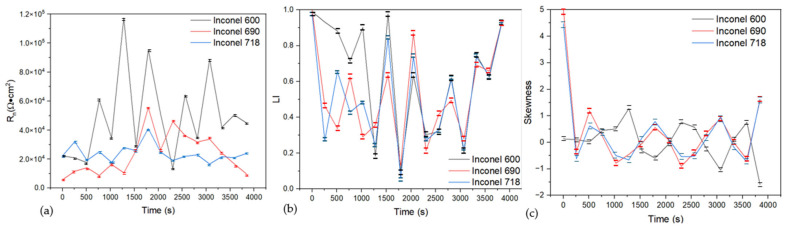
Variation in Rn (**a**), LI (**b**) and skewness (**c**) in different time lapses (each 512 s) in 1 wt. % H_2_SO_4_ solution.

Similar behavior is observed in Figure 6a, where, the R_n_ shows several variations for Inconel 600, indicating more aggressive pitting attacks. When pitting occurs, resistance decreases; if severe, it decreases in several ways.

Figure 6b the LI shows variation, indicating localized corrosion on the material’s surface. On the other hand, the behavior in skewness (Figure 6c) is similar: high values at the first stages. However, after a time, the corrosion falls under 1, indicating that localized attack (pitting) is covering the surface and is beginning to become uniform.

#### 3.2.2. Analysis for Chaotic Systems Such as Hurst, Lyapunov and Husdorff Coefficients

The Hurst exponent (H) has proven particularly effective for quantifying long-range temporal correlations and distinguishing between corrosion modes, such as uniform corrosion, passivation, and localized corrosion (pitting).

The Hurst exponent (H) is calculated using the rescaled range (R/S) analysis. The $\bar{x}$ is the time series (current or potential) of N values, as follows in Equation (5):

(5) $$Y\left(k\right)=\sum_{i=1}^{k}{(x}_{i}-\bar{x}),\;\;\;\;k=1,2,\ldots ,N$$

The range R(n) is the accumulated series over a window of length n defined as Equation (6): R(n) = max [Y(1…n)] − min〖[Y(1…n)]〗

(6) $$\sigma =\sqrt{\frac{1}{n}\sum_{i=1}^{n}{(x}_{i}-{\bar{x})}^{2}}$$

According to Hurst law, the rescaled range follows a power–law relationship (Equation (7)):

(7) $$\frac{R(n)}{\sigma (n)}=Cn^{H}$$

Another method for performing EN analysis is the Lyapunov exponent, which provides a quantitative measure of a system’s sensitivity to initial conditions and allows for the identification of chaotic behavior in time-series data. The application of the Lyapunov exponent to electrochemical noise analysis provides a complementary perspective to fractal and statistical metrics, particularly for characterizing localized corrosion phenomena.

The Lyapunov exponent, denoted by λ, characterizes the average exponential rate at which two initially infinitesimally close trajectories in phase space diverge or converge over time. For a dynamical system, this divergence can be expressed as follows (Equation (8)):

(8) $$d(t)\approx d(0)e^{\lambda \tau }$$

where d(0) s the initial separation between trajectories and d(t) is their separation after time τ. Taking the natural logarithm yields the following (see Equation (9)):

(9) $$\ln\left[d\left(t\right)\right]=\ln\left[d\left(0\right)\right]+\lambda \tau$$

A positive Lyapunov exponent (λ > 0) indicates exponential divergence of trajectories and is a defined signature of chaotic dynamics. A negative Lyapunov exponent corresponds to stable dynamics. At the same time, values close to zero are associated with periodic or quasi-periodic behavior. When applied to electrochemical noise signals, the Lyapunov exponent quantifies the intrinsic dynamical instability of corrosion processes, particularly those dominated by localized and transient events [33,34,35,36].

Electrochemical noise measurements provide scalar time series of current or potential. To compute the Lyapunov exponent, it is first necessary to reconstruct the system’s phase space using time-delay embedding. Given a time series x(i), the reconstructed state vectors are defined as follows (Equation (10)):

(10) $$X\left(i\right)=[x\left(i\right),(x\left(i+\tau \right),\left(x+2\tau \right),\ldots ,x\left(i+\left(m-1\right)\tau \right)]$$

where τ is the time delay and the embedding dimension. The time delay is commonly selected using mutual information analysis. At the same time, the embedding dimension is determined using the false nearest neighbor’s method. Proper selection of these parameters ensures that the reconstructed phase space faithfully represents the underlying corrosion dynamics.

In experimental studies, the largest Lyapunov exponent (LLE) is typically calculated, as it determines whether chaotic behavior is present. The LLE is estimated by monitoring the average divergence of initially close trajectories in the reconstructed phase space. For each reference state vector, the nearest neighbor is identified, and the evolution of the distance between the two trajectories is tracked over time. The average logarithmic divergence is then computed as Equation (11):

(11) $$\ln\left[d\left(t\right)\right]=\lambda \tau +C$$

where C is a constant; the largest Lyapunov exponent λ is obtained as the slope of the linear region in a plot of ln = [d(t)]⟩ versus time τ. A clear linear region with a positive slope indicates chaotic corrosion dynamics, whereas a non-positive slope suggests stable or weakly nonlinear behavior. In electrochemical corrosion studies, the Lyapunov exponent provides direct insight into the nature of the corrosion mechanism:

- λ > 0 indicates chaotic dynamics, commonly associated with localized corrosion processes such as pitting or metastable pit nucleation.
- λ = 0 corresponds to quasi-periodic or transitional regimes between passive and active states.
- λ < 0 reflects stable dynamics, typically observed in uniform corrosion or well-established passive conditions.

The Lyapunov exponent is, therefore, particularly valuable for detecting the early stages of localized corrosion, where chaotic fluctuations may appear in the electrochemical noise signal before visible damage or measurable mass loss occurs [37].

Fractal geometry provides a powerful framework for characterizing such complexity. In particular, the Hausdorff exponent, commonly expressed through the Hausdorff (fractal) dimension, has been widely applied to electrochemical noise analysis to quantify the geometric and dynamical complexity of corrosion processes. The Hausdorff exponent offers insight into the degree of localization and irregularity of corrosion mechanisms, especially in systems prone to pitting and other forms of localized attack.

The Hausdorff dimension H is a measure of how completely a geometrical object or signal fills the space as the scale of observation changes. Unlike Euclidean integer dimensions, the Hausdorff dimension may take non-integer values, reflecting fractal behavior.

For time-series data such as electrochemical noise, the Hausdorff exponent is typically inferred from scaling relationships that describe how a statistical property of the signal varies with observation scale. A general power–law relationship can be written as follows (Equation (12)):

(12) $$M(\epsilon )\propto \epsilon^{D_{H}}$$

where M(ϵ) is the measure of the signal complexity at scale ϵ, and DH is the Hausdorff fractal dimension.

Higher values of DH correspond to more irregular complex signals, while lower values indicate smoother and more regular behavior.

In electrochemical noise analysis, the Hausdorff dimension is estimated using fractal scaling methods applied to time series, such as rescaled range (R/S) analysis, box-counting techniques, or spectral scaling.

For self-affine electrochemical noise signals, the Hausdorff dimension is directly related to the Hurst exponent H through a well-established relationship (see Equation (13)):

(13) $$D_{H}=2-H$$

This relation applies to one-dimensional time series embedded in two-dimensional space (amplitude vs. time) and has been widely adopted in corrosion studies due to its simplicity and robustness [32,38,39,40].

**Figure 7:**
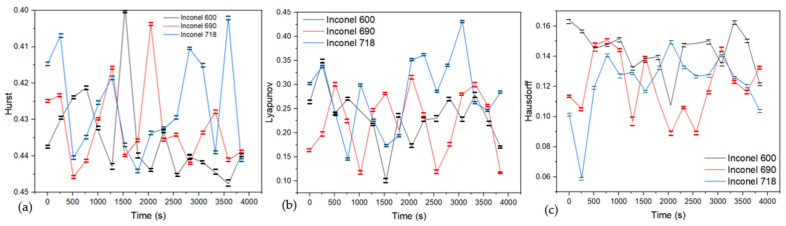
Hurst (**a**), Lyapunov (**b**) and Hausdorff coefficient (**c**) in different time lapses (each 512 s) in 3.5 wt. % NaCl solution.

Figure 7 shows the variation in the Hurst (Figure 7a), Lyapunov (Figure 7b), and Hausdorff coefficients (Figure 7c) at different time lapses (each 512 s) in NaCl. The values obtained by Hurst and Lyapunov are related to anti-persistence, a characteristic of chaotic systems. This occurs due to a localized attack, so, for the values obtained by Hurst and Lyapunov, the corrosion type is localized. On the other hand, Hausdorff is associated with a passive system. However, the microscopy figures do not show that; for this reason, the Hausdorff coefficient is not recommended for analyzing the time series. Values of 0.44 to 0.45 and from 0.15 to 0.4 indicate localized corrosion, potentially related to values from 0.1 to 1 of the localization index, with both results converged in a localized corrosion system.

**Figure 8:**
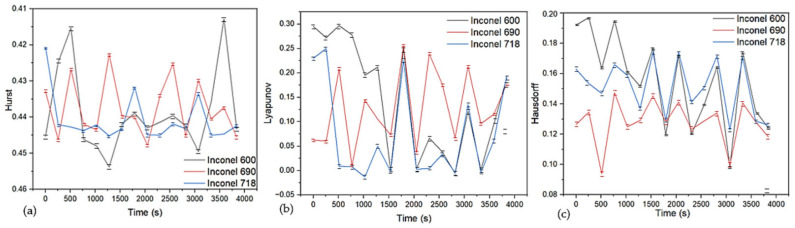
Hurst (**a**), Lyapunov (**b**) and Hausdorff coefficient (**c**) in different time lapses (each 512 s) in 1 wt. % H_2_SO_4_ solution.

Figure 8 shows the variation in the Hurst (Figure 8a), Lyapunov (Figure 8b), and Hausdorff coefficients (Figure 8c) at different time lapses (each 512 s) in H_2_SO_4_. The behavior is very similar to the past, where Hurst and Lyapunov reported values associated with localized corrosion processes, while Hausdorff reported values for uniform and passive systems. It is important to analyze the microscopy from the next section to associate these results with the type of corrosion obtained. That behavior is showed by the values of 0.44 and 0.3 of Hurst and Lyapunov coeficients. The localized index presented values above 0.1, which also indicates localized corrosion.

### 3.3. Cyclic Potentiodynamic Polarization

To validate the electrochemical results, a direct current electrochemical technique, such as cyclic potentiodynamic polarization, was used to identify the type of corrosion of the superalloys in the presence of the electrolytes under study. Electrochemical parameters such as corrosion potential, current density, and pitting potential were also obtained, based on the behavior of anodic and cathodic reactions [41,42]. This method is widely recommended for investigating pitting corrosion in various materials, as specified by ASTM G61 standards [22,23,24,25,26].

**Figure 9:**
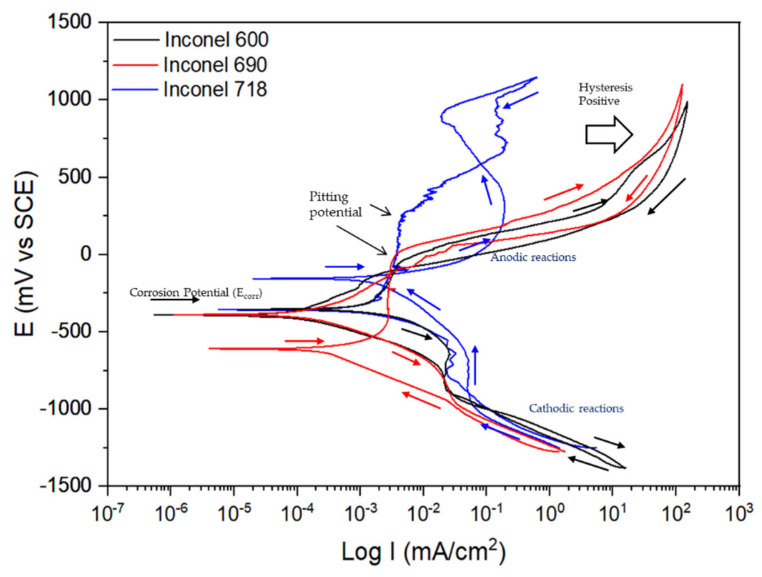
Cyclic potentiodynamic polarization curves for superalloys, exposure to NaCl solution.

Figure 9 presents the polarization curves for the superalloys exposed to a sodium chloride solution. After activation at the anodic branch, both alloys exhibit a pseudo-passivation region, reaching a breakdown or pitting potential (E_pit_) of −15, −61.5 and 257.6 mV, respectively. Inconel 600 and 718 show higher noble values and continue to activate. In the reverse sweep, both alloys exhibit a positive hysteresis loop with a larger area, indicating greater susceptibility to localized pitting corrosion, attributed to the presence of chloride ions.

**Figure 10:**
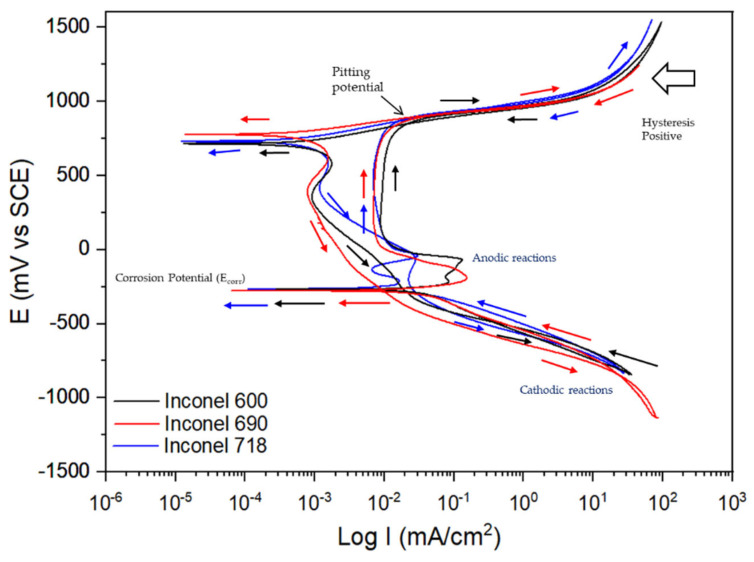
Cyclic potentiodynamic polarization curves for superalloys, exposure to H_2_SO_4_ solution.

Figure 10 shows the curves of the superalloys exposed to the sulfuric acid solution. Both alloys exhibit similar cathodic behavior, and in the anodic branch, a passivation range of 800 mV is observed for all three alloys, followed by transpassivation (breakdown of the passivation film) and a small positive hysteresis loop, indicating slight localized attack in all three superalloys. The corrosion potential (E_corr_) for the three superalloys is −262.11 mV (Inconel 718), −276.34 mV (Inconel 690), and −266.09 mV (Inconel 600), suggesting thermodynamic stability of the superalloys in the presence of the electrolyte (see Table 5). However, the corrosion current densities measured for Inconel 600 and 690 are in the same order of magnitude ×10^−2^ mA·cm^−2^ and x10^−3^ mA·cm^−2^ as Inconel 718, where the lowest current density was found, indicating a reduced corrosion rate.

#### SEM After Electrochemical Noise Measurements

**Figure 11:**
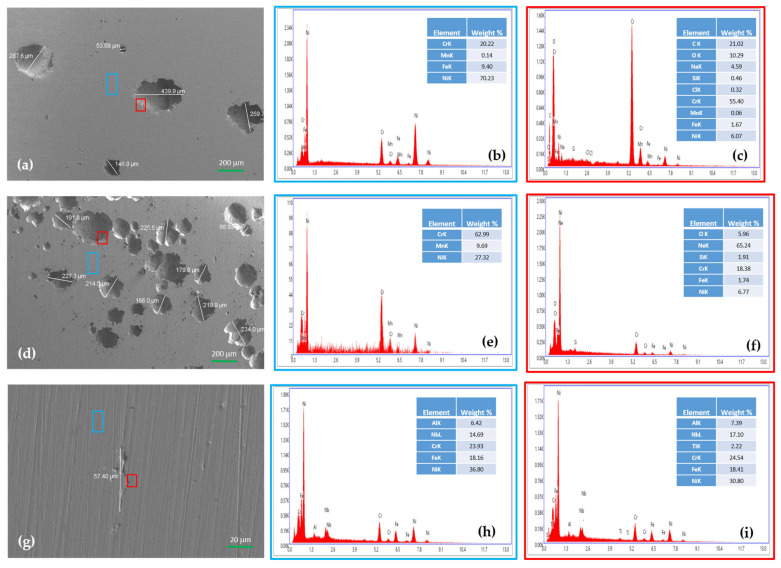
SEM-SE, surface morphology micrographs of superalloy after being exposed in NaCl solution: Inconel 600 (**a**), Inconel 690 (**d**) and Inconel 718 (**g**). EDS spectrum, Inconel 600 (**b**,**c**), Inconel 690 (**e**,**f**) and Inconel 718 (**h**,**i**).

**Figure 12:**
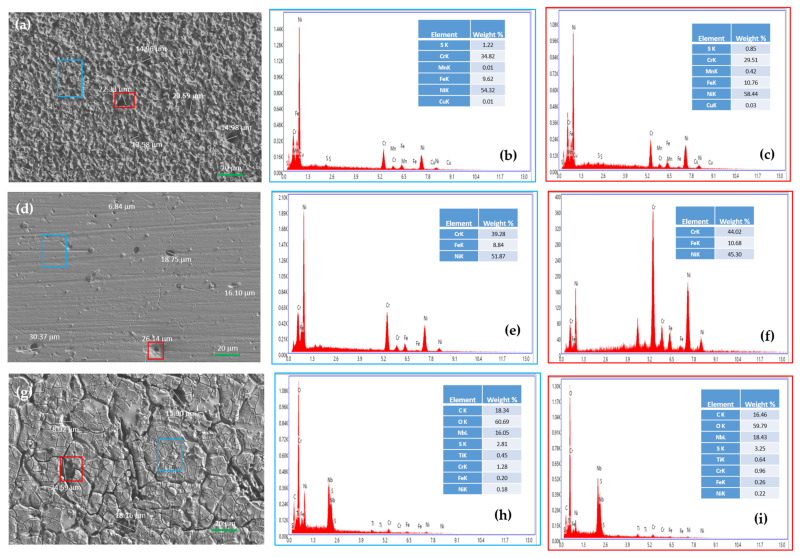
SEM-SE, surface morphology micrographs of superalloy after being exposed in H_2_SO_4_ solution: Inconel 600 (**a**), Inconel 690 (**d**) and Inconel 718 (**g**). EDS spectrum, Inconel 600 (**b**,**c**), Inconel 690 (**e**,**f**) and Inconel 718 (**h**,**i**).

Following electrochemical corrosion measurements, the surface morphology of each sample was examined using SEM and EDS analysis, which revealed localized pitting corrosion on the surfaces of all superalloys (see Figure 11 and Figure 12).

Figure 11 shows a micrographic obtained from the SEM of samples exposed to NaCl solution. Inconel 600 shows pitting corrosion (Figure 11a), where the pits range in size from 53.68 µm to 439.9 µm. In Inconel 690 (Figure 11d), evident localized attack is observed; the pits are more intense, averaging 211.28 µm. However, Inconel 718 also exhibited localized attack but of lesser intensity; therefore, the pitting is smaller, with sizes of approximately 57.40 µm (Figure 11g). The superalloys tested for corrosion have a pitting density of type A (Inconel 600 (A2), Inconel 690 (A3) and Inconel 718 (A1)) according to ASTM G46 [43].

Figure 11b,c,e,f,h,i show EDS spectra of the superalloys exposed in NaCl. Two zones are analyzed: one considered clean (blue box), representing the alloy, and the other a zone with localized etching (red box). The presence of nickel, chromium, iron, and manganese, characteristic elements of the superalloys, is observed in the clean zones of the superalloys. In the area with localized attack (pitting), chlorine, oxygen, and chromium are present (forming chromium oxides). Chemical elements such as niobium, aluminum, and titanium were also present in the Inconel 718 alloy.

Figure 12 shows an SEM micrograph of samples exposed to the H_2_SO_4_ solution. Inconel 600 shows pitting corrosion (Figure 12a), where the pits range in size from 13.58 µm to 22.33 µm. In Inconel 690 (Figure 12d), evident localized attack is observed; the pits are more intense, with an average of 21.092 µm. However, Inconel 718 also exhibited localized attack but of greater intensity than the other superalloys; the pits range from 15.90 to 24.59 µm (Figure 12g). All the superalloys tested for corrosion have a pit density of type A2 according to ASTM G46 [43].

Figure 12b,c,e,f,h,i show the EDS spectra of the superalloys exposed to H_2_SO_4_. Two zones are analyzed: one considered clean (blue box), representing the alloy, and another zone with localized corrosion (red box). In the clean zones of the superalloys, the presence of nickel, chromium, iron, and manganese, characteristic elements of these alloys, is observed. In the zone with localized corrosion (pitting), sulfur, oxygen, iron, nickel, and chromium are present (forming different oxides). In the Inconel 718 alloy, chemical elements such as niobium and sulfur were also detected; some traces of chromium, iron, nickel, and titanium were also observed.

The presence of nickel, chromium, iron, copper, manganese, and silicon is observed in the EDS energy spectrum, corresponding to the base elements of the superalloys under study.

## 4. Discussion

Several studies [30,31,32,44,45,46,47,48] report that various electrochemical noise (EN) techniques, which correlate time-dependent fluctuations in current and potential during corrosion processes, have been used to identify the type of corrosion occurring. It is widely accepted that the main sources of electrochemical noise are the breakdown and subsequent repassivation of the passive film. Understanding these processes is essential for evaluating the behavior of superalloys, as such structural components are exposed to diverse industrial and marine environments.

Most of the available information on low-temperature nickel-based superalloys is based on potentiodynamic polarization techniques and Tafel analysis. In electrochemical noise (EN) measurements, the amplitude of the transients is associated with the size of pit nucleation events, while their frequency is related to localized corrosion activity. Some researchers have attributed these transients to reductions in certain species present in the electrolyte. The transient signals observed in potential and current time series are characteristic of pit nucleation or metastable pitting processes [49,50].

Kup, et al. [51] investigated the effect of alloying elements on the corrosion behavior of several superalloys containing Mo, Ni, and Al using potentiodynamic polarization in a 3 wt.% NaCl solution. Their results showed that the concentrations of Mo, Al, and Ni play a critical role in corrosion inhibition. Linear-sweep voltammetry revealed that the corrosion potential becomes more negative with increasing Mo content. Ting [52] studied the influence of aging treatments on Alloy 718 following different surface finishing processes. The results indicated that surface finishing significantly affects pitting corrosion resistance by altering surface stress conditions.

Electrochemical noise techniques provide mechanistic insight into corrosion systems through statistical analysis. However, authors such as Mansfeld and Eden [32,33] recommend caution when using the localization index (LI) to identify corrosion types. For this reason, the present study employed multiple analytical methods to characterize corrosion behavior. Eden and other researchers proposed using skewness as a statistical parameter for corrosion analysis. Xia et al. [13] reported that these parameters help describe the distribution shape and transient peak characteristics. Skewness helps identify the dominant corrosion process: positive skewness (in ECN data) indicates predominantly anodic transients, whereas negative skewness suggests predominantly cathodic transients. Also, we note that the skewness analysis shows a higher standard error, which can be reduced by increasing the amount of data collected.

The results were consistent with those reported previously and showed good agreement with the localization index (LI) and skewness analyses. Razavi et al. [53] reported that Waspaloy exposed to 3.5 wt.% NaCl under potentiodynamic polarization conditions exhibited pitting corrosion, along with a tendency toward passivation within the potential range of 0 to 473 mV. Similarly, Pan et al. [54] found that the K38C nickel-based superalloy demonstrated passive behavior, although pitting corrosion occurred at higher potentials. These findings agree with the results of the present study and those reported by Razavi, providing further insight into the corrosion behavior of nickel-based superalloys.

However, it is important to note that the use of Hurst, Lyapunov and Hausdorff coefficients is important because each one has different values that classify the corrosion type. Studies have shown that uniform corrosion is typically characterized by H values close to 0.5, indicating weak correlation and quasi-random behavior in current and potential fluctuations [48,55]. Conversely, passive systems and localized corrosion, particularly pitting corrosion, often exhibit H > 0.5, reflecting persistence in the signal due to pit nucleation, growth, and repassivation events [13].

The Lyapunov exponent, originating in nonlinear dynamics and chaos theory, quantifies the sensitivity of a system to initial conditions [17,24,56]. Its application to EN analysis allows the stability of corrosion processes to be evaluated in phase space. Pitting corrosion involves intermittent pit initiation and extinction, resulting in abrupt, irregular current transients. These events increase trajectory divergence in reconstructed phase space, resulting in positive λ values. As a result, the Lyapunov exponent serves as a powerful indicator of electrochemical instability and complements traditional time-domain EN parameters.

The Hausdorff or fractal dimension describes the complexity and irregularity of a signal or surface. In corrosion studies, fractal analysis has been applied to both EN time series and corroded surface morphologies [37,38].

Low fractal dimension values are typically associated with uniform corrosion or stable passivation, where electrochemical reactions proceed smoothly and generate relatively low-amplitude EN fluctuations. In contrast, localized corrosion produces highly irregular signals due to discrete pit nucleation events, resulting in higher fractal dimensions [39,56].

The fractal nature of pitting corrosion reflects its multiscale characteristics, in which microscopic pit initiation events influence macroscopic electrochemical behavior. Therefore, an increased Hausdorff dimension is a strong indicator of corrosion localization.

Although each coefficient provides valuable insight on its own, their combined interpretation significantly strengthens corrosion-type identification. Systems exhibiting a high Hurst exponent, positive Lyapunov exponent, and elevated fractal dimensions are consistently associated with pitting corrosion and other forms of localized attack. Conversely, systems with values close to those of random noise tend to indicate uniform corrosion or stable passive behavior.

This multivariate approach aligns with recommendations by Mansfeld and Eden [30,31,32,57], who emphasized that no single EN parameter should be used in isolation. Instead, corrosion diagnosis should rely on the convergence of multiple statistical and nonlinear indicators. Despite their advantages, nonlinear coefficients require careful application. Reliable estimation of H, λ, and D depends on long, stationary datasets, appropriate sampling frequencies, and proper noise filtering [38,58,59,60]. Furthermore, these coefficients should always be interpreted in conjunction with electrochemical techniques, such as potentiodynamic polarization and surface characterization methods.

Although the present results show a limited correspondence between the estimated fractal (Hausdorff) dimension and corrosion features identified through optical microscopy, this discrepancy should not be interpreted as evidence that the fractal dimension is intrinsically unsuitable for describing corrosion phenomena. The fractal dimension is a statistical parameter that quantifies surface complexity and roughness in a scale-dependent manner, and its physical interpretation strongly depends on image resolution, segmentation procedures, and the effective scaling range considered during analysis [61,62]. In contrast, optical microscopy primarily provides qualitative, mechanism-oriented information and emphasizes localized morphological features, which may not dominate the global scaling behavior of a corroded surface [63].

A quantitative relationship between nonlinear electrochemical noise parameters and localized corrosion susceptibility can be established by analyzing both their magnitude and temporal variability. In the present study, the Hurst exponent, Lyapunov exponent, and Hausdorff dimension were calculated over successive 512 s windows of the EN time series (Figure 7 and Figure 8), allowing each parameter to be evaluated as a time-dependent distribution rather than as a single isolated value. In NaCl solution, Inconel 718 consistently exhibited higher Hurst exponents and positive Lyapunov exponents compared to Inconel 600 and Inconel 690, indicating stronger temporal persistence and greater dynamical instability associated with repeated pit nucleation and repassivation events. In contrast, Inconel 690 showed lower H and λ values with reduced variability, consistent with a more stable passive behavior and higher noise resistance observed in the statistical EN analysis. In H_2_SO_4_ solution, all three superalloys displayed lower dispersion of nonlinear parameters, indicating a less aggressive and more generalized corrosion regime, in agreement with the narrower hysteresis loops observed in CPP curves. The uncertainty associated with nonlinear parameter estimation is reflected in the fluctuations in H, λ, and DH across consecutive time windows; however, the persistence of material-dependent trends throughout the acquisition time indicates acceptable robustness despite the stochastic and non-stationary nature of corrosion processes. It is, therefore, emphasized that these nonlinear coefficients are interpreted in a comparative and trend-based manner, supported by statistical EN parameters and independent electrochemical measurements, rather than as absolute descriptors with fixed threshold values.

Previous studies have demonstrated that different experimental techniques applied to the same corroded material (such as optical microscopy, SEM, holographic microscopy, or electrochemical measurements) can yield distinct fractal dimension values without implying a lack of physical meaning of the parameter itself [64]. Rather, such differences reflect methodological constraints and the inherently multiscale nature of corrosion processes. In this context, the fractal dimension should be regarded as a complementary descriptor of surface morphology, particularly suited for comparative or trend analysis, and not as a standalone indicator of specific corrosion mechanisms. More robust correlations with corrosion evolution are often obtained when fractal metrics are combined with microscopy observations or extended to multifractal and three-dimensional surface analyses [65].

The electrochemical noise response of the investigated nickel-based superalloys can be rationalized through a mechanistic framework that links alloy chemistry, passive film stability, and corrosion dynamics. Alloy composition plays a central role by governing the nature, chemistry, and resilience of the passive film developed on the surface, which in turn determines the dominant corrosion mechanism detected by electrochemical noise analysis [66].

Chromium is the principal element responsible for passivation in Ni-based alloys, promoting the formation of a Cr_2_O_3_-rich inner oxide layer that enhances resistance to uniform corrosion. However, this passive film remains vulnerable to localized breakdown in aggressive environments containing chloride ions. Consequently, alloys with higher chromium content, such as Inconel 690, exhibit greater passive film stability and higher resistance to electrochemical noise, particularly in chloride-containing solutions, reflecting a reduced tendency for spontaneous localized attacks [67].

In contrast, Inconel 718 contains titanium and aluminum additions that promote γ′/γ″ precipitation strengthening. While these precipitates significantly improve mechanical performance, they introduce microstructural and chemical heterogeneities that locally disrupt surface uniformity. These heterogeneities act as preferential sites for passive film rupture, facilitating pit nucleation and growth. This behavior is manifested in the electrochemical noise signals as high-amplitude transients, elevated localization indices, and positive Lyapunov exponents, all indicative of localized, unstable corrosion processes governed by nonlinear dynamics [68,69,70].

The electrolyte chemistry further modulates this response. In chloride-bearing environments, pit stabilization at passive film breakdown sites leads to predominately localized corrosion. In contrast, sulfuric acid environments favor passive film dissolution and repassivation processes, localized initiation coupled with partial surface generalization.

The electrochemical noise (EN) measurements presented in this work were performed under controlled laboratory conditions using static, unstirred electrolyte solutions at room temperature. It is acknowledged that such conditions represent a simplified approximation of real aeronautical service environments. In practice, aircraft components such as turbine blades, landing gear, and structural fasteners experience complex exposure scenarios during ground parking, including cyclic wet–dry conditions, temperature fluctuations, salt deposition, acid rain, and mechanical stresses [39,47,48]. These factors can significantly influence passive film stability and corrosion kinetics, particularly for nickel-based superalloys exposed to marine and industrial atmospheres.

Despite these limitations, static EN immersion tests are widely accepted as a first-order experimental approach for isolating intrinsic corrosion mechanisms, specifically those associated with passive film breakdown, metastable pitting, and repassivation [47,71]. By minimizing external perturbations such as flow, thermal gradients, and mechanical loading, EN measurements under controlled conditions allow direct interpretation of the electrochemical fluctuations originating from localized anodic events. These fundamental processes remain operative in real service environments, although their frequency and severity may be amplified under cyclic or atmospheric exposure conditions.

It is well established that atmospheric corrosion of aeronautical alloys often proceeds through thin electrolyte layers formed during condensation, rainfall, or salt spray, followed by drying periods that concentrate aggressive species such as chloride and sulfate ions. Static immersion tests cannot fully reproduce these cyclic phenomena; therefore, the present results should be interpreted as indicators of material susceptibility to localized corrosion rather than direct predictors of service lifetime. Nevertheless, the electrochemical signatures captured by EN analysis, such as transient amplitude, noise resistance (R_n_), and degree of localization, provide valuable mechanistic insight into corrosion initiation processes relevant to field exposure.

From an applied standpoint, the EN parameters examined in this study offer clear potential for future accelerated testing and field monitoring. Noise resistance (Rn) has been proposed as a non-intrusive indicator of corrosion activity under both laboratory and atmospheric conditions [15,59,72]. Furthermore, nonlinear parameters such as the Hurst exponent and Lyapunov exponent are particularly sensitive to metastable pitting and chaotic electrochemical behavior, making them suitable for detecting early-stage localized corrosion before macroscopic damage becomes evident [6,8]. These descriptors could be incorporated into accelerated corrosion protocols involving cyclic wet–dry exposure, temperature variation, or salt-fog testing, as defined in standardized atmospheric corrosion methodologies [73]. In addition, EN-based sensing approaches have been proposed for in situ corrosion monitoring, offering potential application for condition-based maintenance of aircraft components during ground operation [74].

Overall, while limited to static laboratory conditions, the present EN measurements provide mechanistically relevant information on corrosion initiation in nickel-based superalloys. When interpreted within their experimental limitations and combined with surface characterization, EN parameters constitute a robust foundation for extending corrosion diagnostics toward realistic aeronautical service environments.

Recent advances in electrochemical noise analysis have expanded its applicability beyond classical statistical descriptors and have reinforced the relevance of nonlinear and hybrid signal-processing approaches for corrosion diagnosis. Modern studies have demonstrated that nonlinear parameters, such as the Hurst exponent, Lyapunov exponent, wavelet-based descriptors, and multifractal metrics, provide enhanced sensitivity to metastable pitting events, early passive film degradation, and non-stationary corrosion dynamics that are not adequately captured by conventional EN parameters alone [75,76,77]. In particular, recent works have emphasized the importance of combining time-domain, frequency-domain, and nonlinear analyses to improve the reliability of corrosion mechanism identification, especially for complex alloys and heterogeneous microstructures [77].

Additionally, the recent literature has highlighted the potential of EN-based approaches for real-time monitoring and smart corrosion diagnostics, including machine-learning-assisted signal classification and damage prognosis under variable environmental conditions [78]. These developments support the present study’s methodology and confirm that nonlinear EN analysis remains an active and evolving research area rather than a purely classical technique. By integrating both foundational and recent contributions, the present work positions electrochemical noise analysis as a robust and forward-looking tool for investigating localized corrosion in nickel-based superalloys.

The CPP curves presented above reveal corrosion kinetics of 10^−2^ mA/cm^2^ in NaCl and 10^−3^ mA/cm^2^ in H_2_SO_4_ solutions. Pseudo-passivation is observed in superalloys exposed to sodium chloride, and a passivation range, formed by chromium oxide, is defined for hydrochloric acid. Positive hysteresis is present in all CPP curves, indicating localized pitting attacks and validating the electrochemical noise results.

Upon reaching the breakdown potential, an increase in current density is observed, initiating pit nucleation on the alloy surface. At the end of the reverse scan, the current density decreases due to the reduction in the well growth rate, which ceases upon reaching the repassivation potential, as reported by S. Esmailzadeh et al. [76].

**Figure 13:**
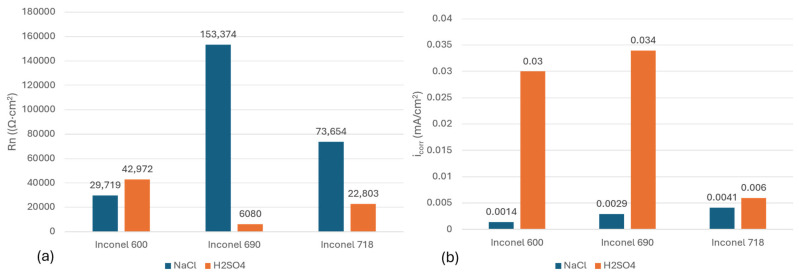
Comparative analysis of electrochemical parameters of superalloy in NaCl and H_2_SO_4_ solutions: (**a**) Rn, and (**b**) i_corr_.

Comparative analysis of corrosion kinetics using electrochemical variables such as electrochemical noise resistance (Rn) and corrosion density (i_corr_), see Figure 13, provides further insight into the systems under study for superalloys exposed to NaCl and H_2_SO_4_ solutions. R_n_ values indicate that higher resistance corresponds to lower current density, analogous to the Ster–Geary equation [29], where R_n_ is inversely proportional to the current density, and, conversely, high current density results in low Rn. Inconel 690 and 718 exhibit the highest Rn values in the presence of sodium chloride, indicating a corrosion density on the order of 10^−3^ mA/cm^2^. Conversely, when Inconel 690 is exposed to sulfuric acid, it shows the lowest Rn values (6080 Ω·cm^2^), and its corrosion density increases to 3.4 × 10^−2^. The same occurs with this alloy in the presence of sodium chloride, having an Rn of 153374 Ω·cm^2^ and a low current density of 2.9 × 10^−3^ mA/cm^2^. Therefore, when a corrosive system such as superalloys in the presence of different electrolytes has very high resistance, it will result in low corrosion densities [27,28,61,78].

Microstructural analyses (OM and SEM) of the superalloys revealed in the EDS spectra that localized corrosion sites (anodic zones) after corrosion tests in the presence of NaCl solution occurred in chromium carbides for Inconel 600 and 690, respectively. In Inconel 690, the anodic sites were found in niobium carbides (NbC), as well as within the austenitic (γ) phase itself. The superalloys, in the presence of H_2_SO_4_ solution, exhibited anodic sites in the austenitic (γ) phase and in chromium carbides (generally Cr_7_C_3_ o Cr_23_C_6_) precipitated at grain boundaries, resulting in high-energy zones and susceptibility to corrosion for Inconel 600 and 690. However, in Inconel 718, SEM and EDS spectra showed the greatest degradation in niobium carbides (NbC), located at grain boundaries when exposed to H_2_SO_4_ solution. Localized corrosion in superalloys occurs mostly in precipitates/carbides found at grain boundaries, causing brittleness in the alloys [79].

Electrochemical characterization of the superalloys could have potential applications in the aeronautical industry, such as aircraft landing gear. The structural components of aircraft made with these steels are exposed to different atmospheres: industrial [acid rain (H_2_SO_4_)] and marine (NaCl). Superalloys may be susceptible to low-temperature localized corrosion when aircraft are on the floor.

## 5. Conclusions

After studying the electrochemical behavior of localized pitting corrosion in superalloys using electrochemical noise analysis in different electrolytes at room temperature, the research results indicate the following:

- The results indicate that superalloys exhibited localized attack in both media; however, NaCl produced more surface pitting.
- Inconel 690 showed higher Rn values in NaCl; however, in H_2_SO_4_, the resistance decreased. For that reason, it is recommendable to use this alloy in salt media.
- The use of methods such as Hurst and Lyanupov is helpful for determining the presence of uniform and localized corrosion. The Hausdorff dimension captures dynamical complexity that is not directly reflected in surface morphology.
- Even though the present results demonstrate only a limited correspondence between the estimated fractal (Hausdorff-type) dimension and corrosion features observed by optical microscopy, this discrepancy does not necessarily indicate that the fractal dimension is fundamentally inappropriate for modeling corrosion behavior.
- The samples treated with H_2_SO_4_ showed localized attacks but not multiple pitting attacks.
- CPP results revealed localized corrosion through hysteresis analysis, with superalloys exposed to NaCl showing the widest loops.

## Figures and Tables

**Table 1:** Chemical composition of different superalloys (wt. %).

Superalloys	Ni	Cr	Fe	Cu	Mn	Si	Nb	Mo	Ti	Al
Inconel 600	72.72 ± 0.06	16.18 ± 0.05	9.10 ± 0.03	0.53 ± 0.02	0.30 ± 0.02	0.22 ± 0.01	0.083 ± 0.002	0.176 ± 0.002	–	–
Inconel 690	61.43 ± 0.06	28.45 ± 0.06	8.23 ± 0.04	0.47 ± 0.02	0.28 ± 0.02	0.47 ± 0.01	0.055 ± 0.001	0.018 ± 0.001	0.38 ± 0.02	–
Inconel 718	50.16 ± 0.07	18.55 ± 0.06	18.65 ± 0.05	0.54 ± 0.02	0.20 ± 0.02	0.25 ± 0.01	4.97 ± 0.01	2.84 ± 0.01	1.03 ± 0.03	0.80 ± 0.07

**Table 2:** Corrosion types are evaluated as skewness [23].

Corrosion Type	Potential	Current
	Skewness	Skewness
Uniform	<±1	<±1
Pitting	<−2	>±2
Transgranular (SCC)	4	−4
Intergranular (SCC #1)	−6.6	1.5 to 3.2
Intergranular (SCC #2)	−2 to −6	3 to 6

**Table 3:** Results obtained from statistical analysis of samples in 3.5 wt. % NaCl solutions.

Sample	R_n_ (Ω·cm^2^)	LI	Corrosion Type	Skewness	Corrosion Type
Inconel 600	29,719 ± 27	0.45 ± 0.02	Localized	1.28 ± 0.07	Localized
Inconel 690	15,3374 ± 58	0.13 ± 0.01	Localized	3.87 ± 0.04	Localized
Inconel 718	73,654 ± 34	0.71 ± 0.02	Localized	13.69 ± 0.02	Localized

**Table 4:** Results obtained from statistical analysis of samples in 1 wt. % H_2_SO_4_ solution.

Sample	R_n_ (Ω·cm^2^)	LI	Corrosion Type	Skewness	Corrosion Type
Inconel 600	42,979 ± 31	0.56 ± 0.01	Localized	−0.28 ± 0.02	Localized
Inconel 690	6080 ± 14	0.25 ± 0.01	Localized	17.15 ± 0.01	Localized
Inconel 718	22,803 ± 24	0.8 ± 0.02	Localized	7.55 ± 0.02	Localized

**Table 5:** Obtained electrochemical parameters for Inconel 600, Inconel 690 and Inconel 718 exposures to NaCl and H_2_SO_4_ solutions.

Solution	Samples	E_corr_ (mV)	i_corr_ (mA/cm^2^)	E_pit_ (mV)	Hysteresis
H_2_SO_4_	Inconel 600	−266 ± 2	3.0 × 10^−2^ ± 0.004	794 ± 4	Positive
	Inconel 690	−276 ± 2	3.4 × 10^−2^ ± 0.001	843 ± 6	Positive
	Inconel 718	−262 ± 3	6.0 × 10^−3^ ± 0.001	819 ± 7	Positive
NaCl	Inconel 600	−352 ± 2	1.4 × 10^−3^ ± 0.002	−61 ± 4	Positive
	Inconel 690	−606 ± 2	2.9 × 10^−3^ ± 0.002	−15 ± 1	Positive
	Inconel 718	−354 ± 2	4.1 × 10^−3^ ± 0.001	257 ± 8	Positive

## Data Availability

The original contributions presented in this study are included in the article. Further inquiries can be directed to the corresponding authors.
